# Potentially Detrimental Effects of Hyperosmolality in Patients Treated for Traumatic Brain Injury

**DOI:** 10.3390/jcm10184141

**Published:** 2021-09-14

**Authors:** Wojciech Dabrowski, Dorota Siwicka-Gieroba, Chiara Robba, Magdalena Bielacz, Joanna Sołek-Pastuszka, Katarzyna Kotfis, Romuald Bohatyrewicz, Andrzej Jaroszyński, Manu L. N. G. Malbrain, Rafael Badenes

**Affiliations:** 1 Department of Anaesthesiology and Intensive Care, Medical University of Lublin, 20-954 Lublin, Poland; manu.malbrain@umlub.pl; 2 Department of Anaesthesia and Intensive Care, Policlinico San Martino, 16100 Genova, Italy; kiarobba@gmail.com; 3 Institute of Tourism and Recreation, State Vocational College of Szymon Szymonowicz, 22-400 Zamosc, Poland; magda.bielacz@gmail.com; 4 Department of Anaesthesiology and Intensive Care, Pomeranian Medical University, 71-252 Szczecin, Poland; pastuszka@mp.pl (J.S.-P.); romuald.bohatyrewicz@pum.edu.pl (R.B.); 5 Department of Anaesthesiology, Intensive Therapy and Acute Intoxications, Pomeranian Medical University, 70-111 Szczecin, Poland; katarzyna.kotfis@pum.edu.pl; 6 Department of Nephrology, Institute of Medical Science, Jan Kochanowski University of Kielce, 25-736 Kielce, Poland; jaroszynskiaj@interia.pl; 7 International Fluid Academy, Dreef 3, 3360 Lovenjoel, Belgium; 8 Medical Department, AZ Jan Palfjin Hospital, Watersportlaan 5, 9000 Gent, Belgium; 9 Department of Anaesthesiology and Intensive Care, Hospital Clìnico Universitario de Valencia, University of Valencia, 46010 Valencia, Spain; rafaelbadenes@gmail.com

**Keywords:** osmolality, traumatic brain injury (TBI), hypertonic saline, mannitol, osmolar gap

## Abstract

Hyperosmotic therapy is commonly used to treat intracranial hypertension in traumatic brain injury patients. Unfortunately, hyperosmolality also affects other organs. An increase in plasma osmolality may impair kidney, cardiac, and immune function, and increase blood–brain barrier permeability. These effects are related not only to the type of hyperosmotic agents, but also to the level of hyperosmolality. The commonly recommended osmolality of 320 mOsm/kg H_2_O seems to be the maximum level, although an increase in plasma osmolality above 310 mOsm/kg H_2_O may already induce cardiac and immune system disorders. The present review focuses on the adverse effects of hyperosmolality on the function of various organs.

## 1. Introduction

Hyperosmotic therapy has been recommended for treatment of cerebral edema (CE) and increased intracranial pressure (ICP) in patients with traumatic brain injury (TBI) and other cerebral diseases [1,2]. The main purpose of increasing the plasma osmolality is to force the shift of water from the brain to the vascular space through the blood–brain barrier (BBB) [2]. According to the Monroe–Kellie doctrine, the sum of the volumes of intracerebral blood, cerebrospinal fluid (CSF), and brain is constant, therefore a decrease of water from the interstitial space of the brain reduces cerebral volume and cerebral edema, which may improve cerebral perfusion [3,4]. Experimental studies have also documented that hyperosmolar therapy attenuates trauma-related inflammatory response by reducing neutrophil activation and neutrophil-endothelium binding [5,6]. Currently, mannitol and hypertonic saline (HTS) have only been recommended for the treatment of intracranial hypertension (ICH) and cerebral edema, and the final goal of hyperosmotic therapy is the achievement of plasma osmolality not higher than 320 mOsm/kg H_2_O [1,2,7]. The choice of agents depends on clinical experience and local protocol, however HTS is frequently used to reduce ICH as well as tissue edema, whereas mannitol is used only to reduce ICH [8,9]. 

## 2. The Most Popular Hyperosmotic Agents

The main problem for choosing hyperosmotic agents is their different osmotic activity. The reflection coefficient (a number which reflects the difficulty for the molecule to pass through the endothelium: 0 = fully permeable and 1.0 = completely impermeable) is 0.9 in the normal brain and a little less in the injured brain [10]. It means that mannitol practically did not pass through the BBB, but it penetrates the injured BBB and the intact BBB. Mannitol at a daily dose of 0.5–1.5 g/kg body weight is commonly used as an osmotically active medication in patients with TBI. Chemically, it is a metabolically inert sugar alcohol (C_6_H_14_O_6_), which is similar to xylitol or sorbitol. It elevates plasma osmolality, which enhances flow from the extravascular to the intravascular space. Interestingly, inhaled mannitol was also indicated by the Food and Drug Administration (FDA) in the treatment of cystic fibrosis in the lung [11,12]. 

HTS elevates plasma osmolality via plasma increase in osmotically active ions, such as sodium. Additionally, HTS also reduces single erythrocyte volume, improving their passage through the capillaries. Its reflection coefficient is 1 [13,14]. It seems to reduce ICH and improve cerebral perfusion pressure more effectively than mannitol [15,16,17]. Some studies also documented better outcomes in patients treated with HTS compared to mannitol, however the osmotherapy-related electrolyte disequilibrium appears to be an independent predictor of poor outcome, regardless of the type of osmotically active medication [18,19,20]. This improves the rheological properties of the blood and the osmotic activity of aquaporin receptors in the BBB [21,22]. Clinicians commonly use HTS with different 3%, 7.5%, or 23.4% solutions, and each of those presents a different osmotic activity (Table 1) [21,22]. Regardless of the type of the osmotically active agents, the main target of osmotherapy is to maintain plasma osmolality around 300–320 mOsm/kg H_2_O [1,2].

## 3. Basic Knowledge

Hyperosmotic therapy is based on osmosis—a phenomenon in which the water molecules migrate through a semi-permeable barrier from a solution rich in osmotically active molecules to a solution poor in the concentration of these agents. The difference in solutes, which cannot pass across the semi-permeable membrane, causes a chemical potential. According to the Gibbs–Duhen equation, the chemical potential and activity of water molecules is higher in a solvent in which the activity of saluted agents is lower, and the movement of water is forced from the solvent to the solution [26]. Osmolarity is defined as the number of solutes per liter of solution, however the concentration of solutes is very low in human body fluids. Therefore, the plasma osmolarity is calculated in milliosmoles (mOsm/L). Osmolality is defined as the number of milliosmoles of solutes per one kilogram of water (mOsm/kg H_2_O). Physiologically, Na^+^, K^+^, Cl^−^, HCO_3_^−^, glucose, and urea are the main osmotically active substances in the human body, however a lot of medicaments exhibit osmotic properties. Some of them, such as urea and ethanol, freely cross the cell membranes and are called “ineffective osmoles”, whereas others such as Na^+^, K^+^, Cl^−^, HCO_3_^−^, and glucose are called effective osmoles because they do not cross the cell membranes, forcing water shifts through the cellular membranes (tonicity). Chemically, osmolarity is strongly related to osmolality in solutions with the same composition but different concentrations of osmotically active agents. These relationship changes occur in the blood because the blood contains lipids, proteins, and others small solutes contributing to plasma osmolality, thus sodium solutions are not completely dissociated in the aqueous medium. Additionally, the plasma contains only 93% of water [27]. Therefore, plasma osmolality can be calculated by multiplying the plasma osmolarity by 0.93. Hence, osmotic pressure is more closely related to plasma osmolality than osmolarity. Plasma osmolality should be measured by a cryoscopy technique, which is considered as the reference method for osmolality measurement [28]. However, several clinicians have calculated plasma osmolality using a different equation. The most popular, the simplest, and the best is known as the Worthley equation [25,27]:

$$Plasma\;osmolality=2xNa^{+}+\frac{Glucose\;\left(mg/dL\right)}{18}+\frac{BUN\;\left(mg/dL\right)}{2.8}=275-295\;mOsm/kg\;H_{2}O$$

**Figure 1:**
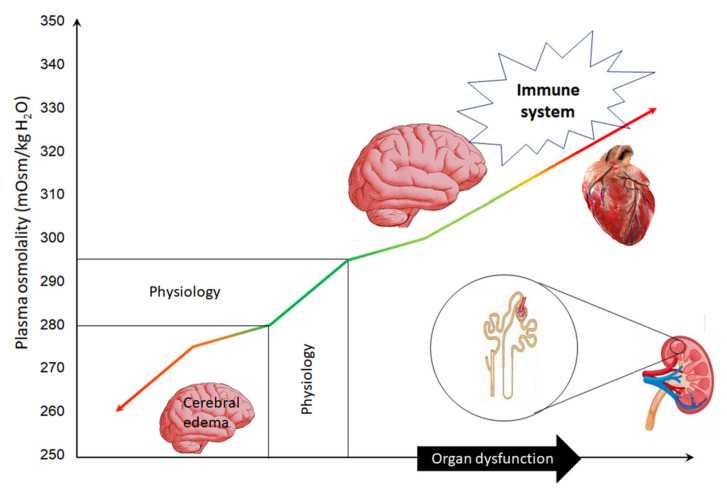
General scheme showing the organs that can be damaged as a result of increased plasma osmolality.

The difference between the measured and the calculated plasma osmolality is called the osmolal gap. Physiologically, its value ranges between −10 and +10 mOsm/L. An osmolal gap higher than 10 mOsm/L documents the presence of osmotically active agents in the blood, while its values above 20 mOsm/L suggest blood intoxication with strong osmotic substances [29,30,31]. Despite the beneficial effect of elevated plasma osmolality on cerebral water content in TBI patients with cerebral edema, hyperosmolality per se or associated with high osmolal gap may affect organ function, increase the risk of multiorgan dysfunction, and worsen the outcome in critically ill patients (Figure 1) [30,31,32,33,34]. The aim of this article was to provide a narrative review regarding the effect of recommended plasma hyperosmolality on organ function in patients treated for TBI.

## 4. Plasma Hyperosmolality and the Heart

The disorders of plasma osmolality can impair cardiac function and increase the risk of life-threatening cardiac arrhythmias and sudden cardiac death [32,35,36,37,38]. An analysis of relationships between plasma osmolality, and the 30-day and 1-year outcomes in 985 patients diagnosed with acute coronary syndrome, showed a significantly higher mortality rate in patients with hyperosmolal plasma [38]. Another clinical analysis of 3748 patients treated for acute coronary diseases also documented an increase in short and long mortality in patients with hyperosmolality [36]. Interestingly, the rate of ventricular arrhythmias, cardiogenic shock, and major adverse cardiac events was two-fold higher in those patients. Indeed, an increase in plasma osmolality following mannitol administration above 313 mOsm/kg H_2_O significantly increased the risk for prolongation of corrected QT interval above 500 ms, which is associated with the incidence of atrial fibrillation in patients without any cardiac history treated for TBI [32]. An experimental study has shown that HTS-induced hyperosmolality per se may exert potentially deleterious effects on myocardial contractility, leading to systolic and diastolic dysfunction, cytosolic Ca^2+^ accumulation with diastolic contracture, and increased susceptibility to life-threatening arrhythmias [27]. Additionally, HTS-related hyperosmotic stress is associated with an increase in the intracellular Ca^2+^ concentration and generation of reactive oxygen species, which promotes stress in the endoplasmic reticulum, leading to apoptosis and death of adult and neonatal cardiomyocytes [39,40]. Plasma osmolality plays a crucial role in the function of cardiac aquaporins. Hyperosmolality increases the mRNA of aquaporin-1, mRNA of upregulated aquaporin-7, protein glycosylation, and intracellular translocation, which may modulate water transport in cardiac myocytes [41,42,43]. A rapid increase in plasma osmolality following hypertonic saline administration depresses the sensitivity of the cardiac baroreflex independently of changes in blood pressure, causing an increase in heart rate [44]. Accumulating data have shown that a rapid increase in plasma osmolality activates sympathetic nerve activity, both in humans and animals [45,46,47]. Moreover, prolonged hyperosmolality also increases sympathetic nerve activity through activation of osmoreceptors and raised excitatory amino acid release in the forebrain [47,48]. A dysregulation of sympathetic/parasympathetic activity as well as dysfunction of cardiac myocytes following an increase in plasma osmolality may depress cardiac function, leading to acute cardiac failure. Thus, it can be speculated that hyperosmolality may play an important role in cardiac dysfunction that develops in patients treated for TBI, which is commonly known as the brain–heart interaction.

**Figure 2:**
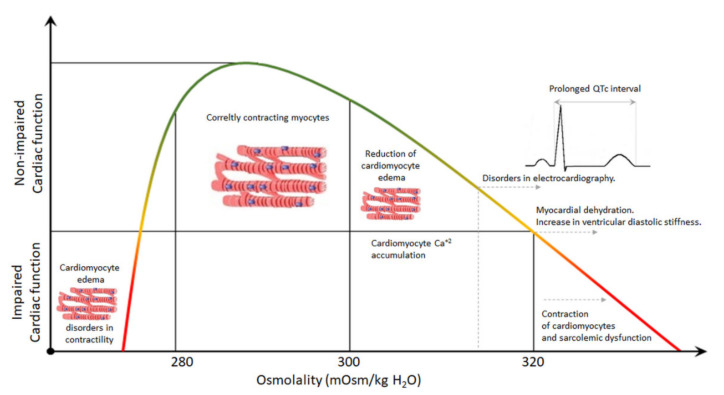
General scheme showing the effect of hyperosmolality on the heart.

In some clinical situations, hyperosmolality may also have a beneficial effect on cardiac function. Experimental studies documented that hyperosmotic perfusion significantly reduced total and intracellular myocardial water content, reduced sarcolemmal rupture, and increased coronary flow in ischemia/reperfusion-induced cellular edema [49,50]. Another study documented that hyperosmotic pretreatment also reduced the infarct size following regional-induced ischemia in a rat heart model [51]. The beneficial effect of hyperosmotic perfusion after cardiac ischemia may be explained by the relatively small osmotic gradient between the intra- and extra-cellular spaces during reperfusion. An increase of the level of intracellular lactate following ischemia-induced anaerobic glycolysis results in a relative hyperosmotic condition within the ischemic area. Hence, the normo- or hypo-osmotic reperfusion increases the water shift from the vascular into the intracellular space, leading to cellular edema, whereas hyperosmotic reperfusion does not induce water extravasation (Figure 2). It is also worth stressing that a lot of research analyzing the beneficial effect of hyperosmolal reperfusion in ischemic heart with swollen cardiomyocytes showed that the increased osmolality of the perfusate (with mannitol) had cardioprotective properties [52]. Taken together, we can suggest that hyperosmolality may impair cardiac function in TBI patients without any previous history of cardiac diseases. Hence, osmotherapy requires strict control of plasma osmolality (not osmolarity).

## 5. Plasma Hyperosmolality and the Kidney

**Figure 3:**
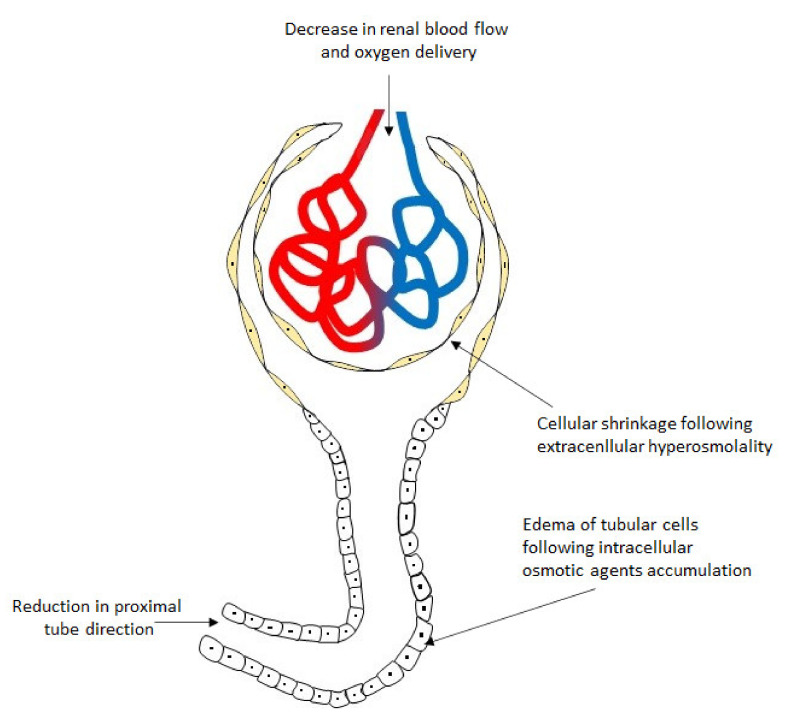
General scheme presenting an effect of hyperosmosis on glomerulus and tubular cells [54,55,56,57,66].

Kidneys are especially vulnerable to disorders in plasma osmolality because they play a crucial role in plasma osmolality regulation. The kidney is responsible for regulation of salt and water excretion. Under physiological conditions, sodium is the predominant cation affecting fluid osmolality in mammals, and the osmoregulation and the control of total body sodium operate independently to its plasma concentration, at least to some extent [53]. Several factors play a role in the regulation of kidney excretory function, and inner medullary cells are especially vulnerable to elevation of plasma osmolality. Hyperosmolality induces salt excretion, increasing its concentration in urea and inner medullary cells. This process forces increased urea removal. It is noteworthy that Na^+^ and Cl^−^ exert different effects on cells due to their different permeability of the cell membranes, whereas urea penetrates the cell membrane similarly to water. Extracellular hypertonicity following elevated extracellular salt content increases passive water shift from the intracellular into the extracellular space, leading to cellular shrinkage. On the other hand, elevated urea concentration in the extracellular space forces its shift to the inner medullary cells due to osmosis. Accumulated intracellular urea is a trigger for uncontrolled protein denaturation. Additionally, the nonspecific effect of hyperosmolality may result from osmolar-forced diuresis with activation of tubulo-glomerular feedback associated with an increase in hydrostatic pressure in the tubules and a decrease in intrarenal microcirculation flow, which ultimately reduces the glomerular filtration rate. An impairment of renal blood flow disturbs oxygen delivery to the renal cells, inducing hypoxia-related cell damage [54]. Hence, hyperosmolality itself affects cell volume, cell metabolism, intracellular ion homeostasis, and stability of nucleic acids, which can induce an apoptotic process and upregulate several genes in the renal inner medullary cells [55,56,57,58]. A lot of osmotically active agents may also induce or intensify hyperosmosis-related acute kidney injury (AKI). This pathology is commonly known as “osmotic nephrosis” or “sucrose nephrosis” (Figure 3). Several studies showed that intravenous administration of immune globulin, mannitol, contrast media, hydroxyethyl starch solutions, or glucose can induce AKI injury via osmotic cell destruction [59,60,61,62,63,64,65]. It was well-documented that osmotically active agents entered the tubular cells by means of pinocytosis, leading to cellular edema with increased lysosomes and endocytotic vacuoles. Interestingly, the use of iso-osmolar contrast media also results in nephrotoxicity, similar to the effect of the hyperosmolar media, which cannot be explained by hyperosmolality itself, but rather the increased viscosity of the iso-osmolar agents [65,66]. However, plasma osmolality plays an important role for renal function. Clinical observations documented a significant relationship between plasma osmolality and a higher incidence of AKI noted in patients with diabetic ketoacidosis when osmolality exceeded 320 mOsm/kg [67,68]. The osmotic nephrosis is usually reversible after discontinuation of osmotically active agents; however, some patients require temporary renal replacement therapy [63,68,69,70,71].

Mannitol is not recommended for use in the management of severe TBI when ICP and brain tissue oxygen are monitored [2]. Several studies documented AKI following mannitol administration [62,69,70,71]. Mannitol-induced osmotic nephrosis has been well-documented, as it exerts nephrotoxic activity [61,62,63,65,72]. There is a dose–response relationship between the use of mannitol and the incidence and severity of AKI, with a cut-off of the daily dose at 1.34 g/kg body weight [73]. Interestingly, the combined therapy of ICH with mannitol and HTS did not increase the risk of AKI more than HTS alone, however several authors suggested to use HTS, demonstrating its superiority over mannitol [13,14,16,18,74]. In conclusion, it can be postulated that an increase in plasma hyperosmolality per se, as well as the use of osmotically active medications, may impair renal function, and that maintaining adequate renal perfusion may reduce the risk of AKI.

## 6. Plasma Hyperosmolality and Immune System

The effects of hyperosmolality on the immune system are still controversial and not very well-recognized, however several in vitro studies have attributed an important role to hypertonicity in the inflammatory response [75,76,77,78,79,80]. Elevated plasma osmolality is especially associated with stimulation of macrophages and dendric cells [5,75]. An increase in plasma osmolality by 10 to 20 mOsm/kg suppresses neutrophil function by modulating cellular signaling, fosters B cell activation and differentiation, and reduces macrophage activation [5,76,77,78]. Several experimental studies have documented that increasing tonicity inhibits the production of proinflammatory cytokines in pulmonary epithelial cells [78,79]. The inhibitory effect of hypertonicity on inflammatory responses is especially important after brain injury. An increase in plasma osmolality following mannitol or HTS administration reduces microglial activation and promotes the anti-inflammatory phagocytic M2-like microglial phenotype in an experimental model of intracerebral hemorrhage [5]. Such relationships between hyperosmolality and the inflammatory response may result from direct regulation of nuclear factor in the T cells, which affect TNF-α and lymphotoxin-β [80]. Additionally, hyperosmotic stress leads to cell apoptosis that involves changes in the apoptotic signaling molecules such as mitogen-activated protein kinase, c-Jun amino terminal kinase, mitogen-activated kinase, and p38 mitogen-activated kinase in a primary cultured nucleus pulpous cells [81]. Hyperosmolarity following mannitol administration at the dose of 1.0–1.5 g/kg body weight induces programmed cell death in a dose-dependent manner in both endothelial and smooth muscle cells [82]. The cell loss within the endothelial monolayers was the most pronounced, with serum osmolarity above 320 mOsm/L. Quite the opposite, it has been documented that hyperosmotic stress is associated with pro-inflammatory cytokine secretion, such as: TNF, IL1-β, IL-6, and IL-8, and that hyperosmolality may be an important factor for survival of macrophages at the inflammatory site after injection of the Bacille Calmette-Guerin (BCG) vaccine [83]. Additionally, prolonged dietary sodium administration increases activation of stress-sensitive neurons of the hypothalamic paraventricular nucleus and basolateral amygdala, leading to stress coping behaviors in mice [84]. In a clinical study including 44 healthy volunteers who received a 250 mL intravenous bolus of 3% saline solution to increase plasma osmolality to 315 mOsm/L, the authors showed that both hyponatremia and plasma hyperosmolality did not induce an increase in circulating markers of inflammation and led to a decrease in the level of TNFα and IL-8 at an unchanged level of IL-6 plasma concentration [85]. Another study documented that the increase in plasma osmolality following mannitol at a dose of 0.5 g/kg body weight significantly limited cardiopulmonary bypass-related inflammatory response, with a reduction of pro-inflammatory and an increase of anti-inflammatory cytokines [86]. It is noteworthy that the majority of studies analyzing the effects of hyperosmolality on the immune system are based on experimental observations. Therefore, one can only speculate that hyperosmolality seems to have a beneficial effect on the immune system, and this hypothesis should be confirmed in further studies.

## 7. Plasma Hyperosmolality and the Blood–Brain Barrier

Hyperosmolar therapy is the cornerstone treatment of ICH. Administration of hyperosmolar agents increases the osmotic gradient between blood and brain, forcing the water flux from the brain to blood through the BBB. In the central nervous system of mammals, the BBB is created at the level of the endothelial brain cells, where multiple protein complexes accumulate at the cell-junctions, restricting the paracellular diffusion of ions and other polar solutes, hence effectively blocking the penetration of macromolecules. Unfortunately, therapeutic hyperosmolar agents can reversibly open thigh junctions in the cerebrovascular endothelium, and their conductivity depends on the degree of plasma hyperosmolality [87,88,89,90]. An experimental study has shown a temporal induction of neuroinflammatory response following intracarotid infusion of mannitol [89]. Elevation of cytokines, chemokines, trophic factors, and cell adhesion molecules was noted within 5 min after mannitol administration that persisted for 4 days. It is noteworthy that the BBB’s susceptibility to increase plasma osmolality decreases with age and is the greatest in fetuses and premature infants [90].

**Figure 4:**
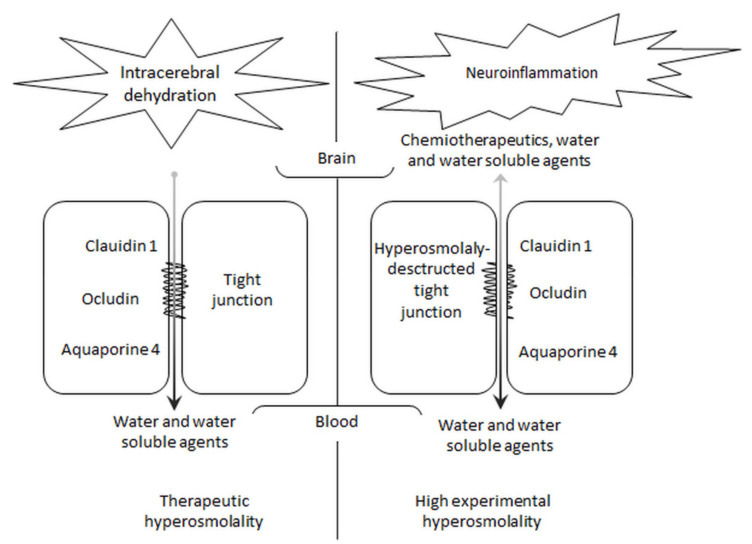
General scheme showing the effect of hyperosmolality on the blood–brain barrier. Therapeutic increase in plasma osmolality intense water removal from the brain. Experimentally raised osmolality to the high value disrupts the blood–brain barrier from opening the tight junction for intracerebral shifts of chemotherapeutics, water, and other water-soluble and insoluble agents.

Currently, the effect of a rapid increase of plasma osmolality on the function of the BBB is used to increase delivery of poorly penetrating medications to the brain (Figure 4). This type of treatment may be especially attractive for treating malignant brain tumors [91,92]. Administration of a small volume of chemotherapeutics after mannitol into the tumor circulation increases their therapeutic properties without the need for increased systemic doses and without adverse effects [91]. A lot of preclinical and clinical studies have convincingly documented the high potency of this approach to elevate the delivery of chemotherapy and other medications to the brain. Experimental studies have also presented a better brain delivery of other drugs, such as antiepileptic drugs or docosahexaenoic acid (DHA), in hypertonicity-related hyperpermeability of the BBB [93,94]. Interestingly, an increase of DHA attenuates BBB disruption, and reduces cerebral edema and TBI-induced neuroinflammation [94,95].

It is difficult to show a destructive effect of plasma hyperosmolality on the BBB in patients treated for TBI. An experimental and therapeutic decrease in BBB permeability is induced by intra-arterial administration of mannitol. Hence, many clinicians prefer HTS over mannitol to increase plasma osmolality, because HTS does not affect the BBB permeability. However, a decrease in BBB permeability following hypertonicity seems to be useful in treating secondary brain damage from different antioxidants and anti-inflammatory agents. This hypothesis needs confirmation in future studies.

## 8. Conclusions

Osmotherapy is the cornerstone treatment of ICH. An increase in plasma osmolality to the recommended 320 mOsm/kg H_2_O is commonly achieved by mannitol or HTS. The choice of osmotic agents is still the subject of debate, and HTS seems to be preferred over mannitol. An increase in plasma osmolality may impair cardiac, kidney, immune, and BBB function, however a deleterious effect of mannitol-induced hyperosmolality has only been clinically documented with respect to kidney and cardiac function. An increase in plasma osmolality per se above 313 mOsm/kg H_2_O may by itself impair cardiac function. Future trials are awaited to bring more answers and solutions.

## Figures and Tables

**Table 1:** Theoretical osmolality of the most popular osmotically active agents [17,18,19,20,21,22,23,24,25].

Solution	0.9% NaCl	3% NaCl	7.5% NaCl	23.4% NaCl	10% Mannitol	15% Mannitol	20% Mannitol	1‰ Ethanol
Osmolality (mOsm/kg H_2_O)	308	1026	2567	8008	550	825	1100	22

## Data Availability

Not applicable—not original data.
